# Sirénomélie (Mermaid Syndrome): description du premier cas Congolais et revue de la literature

**DOI:** 10.11604/pamj.2014.17.162.3934

**Published:** 2014-03-06

**Authors:** Toni Kasole Lubala, Olivier Mukuku, Augustin Mulangu Mutombo

**Affiliations:** 1 Département de Pédiatrie, Faculté de Médecine, Université de Lubumbashi, République Démocratique du Congo

**Keywords:** Mermaid syndrome, sirénomélie, dysgénésie caudale, Mermaid syndrome, sirenomelia, caudal dysgenesis

## Abstract

La sirénomélie est une forme rare de dysgénésie caudale généralement incompatible avec la vie du fait des malformations rénales graves qui y sont associées. En Afrique, elle est associée à des considérations mystico-religieuses et à la sorcellerie et expose la famille à une stigmatisation violente. Son étiologie est encore très controversée. A notre connaissance, il s'agit du premier cas congolais rapporté dans la littérature.

## Introduction

La sirénomélie est une forme rare de dysgénésie caudale décrite pour la première fois par Rocheus en 1542 puis par Polfyr en 1553 [1]. Elle est caractérisée par un degré variable de fusion des membres inférieurs. Sa prévalence est estimée à 1 pour 100000 naissances vivantes [2]. Plus de 50% des patients décèdent in utéro, et ceux qui naissent vivants décèdent généralement dans les 48 premières heures de vie du fait des graves malformations rénales fréquemment associées à cette séquence. En Afrique, la naissance de ces nouveau-nés dit « enfants poissons » est souvent associée à des considérations mystico-religieuses et les mères de ces enfants souvent accusées de sorcellerie. Très peu de cas ont été rapportés en Afrique [3, 4]. Nous rapportons le premier cas congolais de sirénomélie, observé à Lubumbashi, dans le sud-Est de la République Démocratique du Congo.

## Patient et observation

Madame D.M, Congolaise âgée de 26 ans, P6G7A0D0, à 36 semaines d'aménorrhée consulte pour hydrorrhée et douleur d'enfantement. Par ailleurs, elle signale la non perception de mouvements foetaux actifs il y a quelques heures. Tout au long de sa grossesse, elle n'a suivi aucune consultation prénatale et n'a pris aucun médicament ni aucun produit traditionnel. Elle n'est pas diabétique et n'a aucun antécédent personnel particulier. Aucune notion de diabète ni de malformations n'est retrouvée dans sa famille. Pas de consanguinité avec son conjoint âgé de 41 ans.

**Figure 1:**
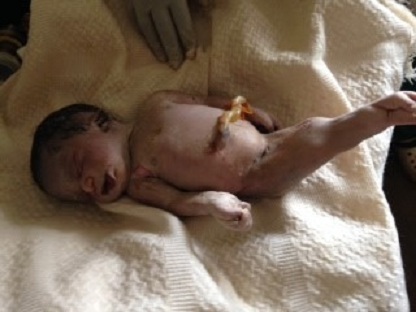
Vue antérieure

**Figure 2:**
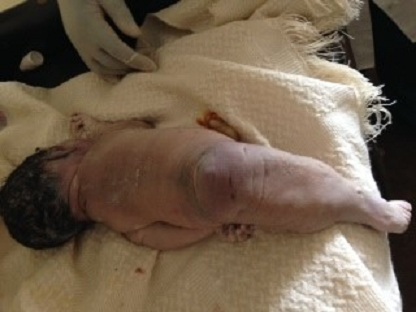
Vue postérieure montrant l'imperforation anale

**Figure 3:**
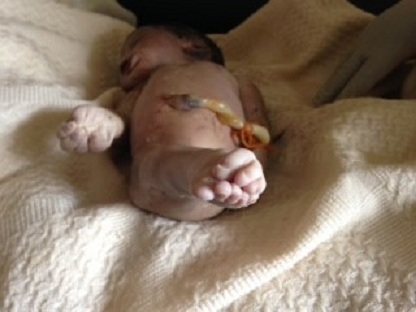
Vue inférieure montrant les 9 orteils

A l'admission, l'état général était bon et les signes vitaux dans les normes. La hauteur utérine était de 32 cm et l'auscultation au foetoscope de Pinard notait l'absence de bruits du coeur foetal. Le foetus était en présentation du siège, le toucher vaginal mettait en évidence l'absence de membranes foetales et permettait de palper les membres inférieurs f'taux qui étaient collés. La patiente fut mise sous antibioprophylaxie et nous avons laissé évoluer le travail d'accouchement. Au bout de 4 heures, elle accoucha un mort-né macéré au premier degré ayant pesé 3000 grammes et mesuré 47 cm de taille et 33,5 cm de périmètre crânien. L'examen du nouveau-né notait la présence d'une artère ombilicale unique, l'absence des organes génitaux externes (Figure 1), une imperforation anale (Figure 2) et des membres inférieurs fusionnés de leur base jusqu'aux pieds (Figure 1, Figure 2, Figure 3) et leur palpation externe donnait l'impression d'avoir probablement deux fémurs et deux tibias. Les deux pieds étaient reliés par leurs plantes avec 9 orteils repartis sur deux lignes (Figure 3). Le nouveau-né avait l'aspect d'une « sirène ». Nous n'avons pas pu obtenir le consentement des parents afin de réaliser des radiographies des membres inférieurs chez le mort-né et des dosages sanguins des métaux lourds ainsi que la glycémie chez la mère.

## Discussion

L'étiologie de la sirénomélie est encore controversée. Généralement sporadique, elle a été longtemps considérée comme d'origine environnementale, avec le diabète maternel insulino-dépendant comme première cause compte tenu du fait que 10 à 15% des enfants affectés sont nés de mères diabétiques. Certains auteurs ont suggéré que le stress oxydatif induit par l'accumulation de radicaux libres pourrait avoir un effet tératogène chez les foetus de mères diabétiques. Cependant, seuls 0,5%-3,7% des cas rapportés dans la littérature ont été associés à un diabète maternel. Celui-ci n'explique donc pas à lui seul la survenue de cette séquence malformative. Dans notre observation, la mère n'était pas diabétique. Cependant, la grossesse n'ayant pas été suivie dans le cadre des consultations prénatales, il ne nous est pas possible d'exclure formellement le diabète gestationnel. D'autres facteurs tératogènes tels que la vitamine A, le Cadmium, le plomb ont été associés à la sirénomélie sur des modèles animaux et humains. Etant donné qu'il a été trouvé des taux élevés de cadmium et de plomb dans la population lushoise [5], l'hypothèse étiologique impliquant ces métaux lourds n'est pas à écarter.

En 1986, Stevenson et al ont proposé une théorie du « vol vasculaire » dans laquelle la régression caudale résulterait d'un détournement du flux sanguin destiné à la partie caudale de l'embryon via un large vaisseau dérivant de l'artère vitelline prenant son origine dans l'aorte abdominale [6]. Ce détournement entraine une hypoperfusion du mésoderme caudal. Cependant, il convient de souligner le fait que cette théorie n'explique pas, à elle seule les cas associant des malformations crâniofaciales et cardiaques. De plus, elle ne donne pas la cause de cette séquence malformative.

La théorie du défaut de la blastogenèse a également été proposée pour expliquer la pathogenèse de la sirénomélie. Selon cette théorie, la sirénomélie est une anomalie primaire de la blastogenèse qui survient à la fin de la gastrulation vers la troisième semaine de gestation. En 1961, Duhamel et al ont observé une association fréquente de la sirénomélie avec des malformations de la région caudale de l'embryon. (anorectales, génito-urinaires, lombo-sacrées,…), définissant un ensemble malformatif qu'il a dénommé syndrome de régression caudale mais aujourd'hui connu sous le nom de dysgénésie caudale. Il a été récemment suggéré que les deux hypothèses demeurent valables, les anomalies de la blastogenèse pouvant affecter tant les organes que les vaisseaux [7].

La publication récente de cas familiaux a stimulé la recherche de facteurs génétiques associés à la sirénomélie [8]. Des études expérimentales réalisées chez des rats induisant des mutations perte de fonction des séquences de signalisation du gène « Bone morphogenetic protein » (BMP-7) ou des mutations gain de fonction des séquences de signalisation du gène «retinoic acid » (RA) ont permis d'observer le développement d'un phénotype semblable à celui observé chez l'humain, associant la fusion des membres inférieurs à des malformations pelviennes sévères [9]. Il semble donc qu'il existe une base génétique complexe, polygénique dans la pathogenèse de la sirénomélie.

Dans le cas de notre patient, aucune échographie n'a été réalisée dans le cadre des consultations prénatales. Pourtant, le diagnostic échographique précoce est possible dès la 9ème semaine de grossesse en dépit de l'hydramnios sévère consécutif à l'agénésie rénale bilatérale observée dans la grande majorité des cas [10].

## Conclusion

La sirénomélie est une malformation congénitale rare et fatale. A notre connaissance, il s'agit du premier cas documenté en République Démocratique du Congo. Le refus par les parents de réaliser des examens complémentaires tant à la mère qu'à l'enfant a considérablement limité notre description clinique.
